# Using fNIRS to evaluate ADHD medication effects on neuronal activity: A systematic literature review

**DOI:** 10.3389/fnimg.2023.1083036

**Published:** 2023-01-24

**Authors:** Eva Poliakova, Amy L. Conrad, Kelly M. Schieltz, Matthew J. O'Brien

**Affiliations:** ^1^Stead Family Department of Pediatrics, The University of Iowa, Iowa City, IA, United States; ^2^Carver College of Medicine, The University of Iowa, Iowa City, IA, United States

**Keywords:** fNIRS, ADHD, medication, children, adolescents, systematic literature review

## Abstract

**Background:**

Functional near infrared spectroscopy (fNIRS) is a relatively non-invasive and inexpensive functional neuroimaging technique that has shown promise as a method for understanding the differences in neuronal activity associated with various neurodevelopmental conditions, including ADHD. Additionally, fNIRS has been suggested as a possible tool to understand the impact of psychotropic medications on brain activity in individuals with ADHD, but this approach is still in its infancy.

**Objective:**

The purpose of this systematic literature review was to synthesize the extant research literature on the use of fNIRS to assess the effects of ADHD medications on brain activity in children and adolescents with ADHD.

**Methods:**

A literature search following Preferred Reporting Items for Systematic Literature Reviews and Meta-Analyses (PRISMA) guidelines was conducted for peer-reviewed articles related to ADHD, medication, and fNIRS in PsychInfo, Scopus, and PubMed electronic databases.

**Results:**

The search yielded 23 published studies meeting inclusion criteria. There was a high degree of heterogeneity in terms of the research methodology and procedures, which is explained in part by the distinct goals and approaches of the studies reviewed. However, there was also relative consistency in outcomes among a select group of studies that demonstrated a similar research focus.

**Conclusion:**

Although fNIRS has great potential to further our understanding of the effects of ADHD medications on the neuronal activity of children and adolescents with ADHD, the current research base is still relatively small and there are limitations and methodological inconsistencies that should be addressed in future studies.

## Introduction

Attention deficit/hyperactivity disorder (ADHD) is among the most common mental disorders among children and adolescents in the U.S. with an estimated prevalence of 6 million (Bitsko et al., 2022). ADHD is a neurodevelopmental disorder with hallmark symptoms of hyperactivity, impulsivity, and inattentiveness (American Psychiatric Association., 2013). Although the etiology of ADHD is not completely understood, research points to a combination of genetic and environmental contributors (Faraone et al., 2021). Specific risk genes have been identified (Liu et al., 2017; Grünblatt et al., 2019; Bonvicini et al., 2020) and environmental exposure to lead (Nilsen and Tulve, 2020), *in utero* maternal smoking (Nilsen and Tulve, 2020), and very preterm birth (Franz et al., 2018) have been associated with ADHD. Studies of ADHD in children have identified some neuroanatomical differences in individuals with ADHD, including lower total cerebral volume and changes in the frontal and parietal lobe, basal nuclei, globus pallidus, corpus callosum and cerebellum (Vieira de Melo et al., 2018). More recently, research has turned its focus to understanding ADHD in childhood based on neural network dysfunction. For example, studies have shown hypoactivation of the right inferior frontal cortex, supplementary motor area and basal ganglia (Samea et al., 2019; Lukito et al., 2020).

More than three-fourths of U.S. children with ADHD receive behavioral therapy, pharmacological treatment, or both (Danielson et al., 2018). The majority of children with ADHD (62%) are treated medicinally. Medications approved by the U.S. Food and Drug Administration to treat ADHD include short- and long-acting stimulants and antihypertensives, and a selective norepinephrine reuptake inhibitor (i.e., atomoxetine). These medications may be delivered orally *via* pill, liquid, chewable tablet, or transdermally *via* patch.

Stimulants, including amphetamine derivatives (e.g., lisdexamfetamine, mixed amphetamine salts) and methylphenidate derivatives, are currently the first-line drugs approved for alleviating ADHD symptoms (Cortese et al., 2018) and effects can last up to 3 to 5 h (for short-acting stimulants) or 8 to 12 h (for long-acting stimulants). The neurochemical mechanisms underlying the effect of stimulants have not been fully determined, however, studies have shown that methylphenidate inhibits dopamine and norepinephrine transporters, which are responsible for modulating sufficient reuptake of neurotransmitters (Makris et al., 2009; Berridge and Devilbiss, 2011). One of the main neuropharmacological effects of ADHD stimulants is an increase in norepinephrine and dopamine levels in the prefrontal cortex and striatum, thereby improving executive function and alleviating ADHD symptoms (Arnsten, 2006). Stimulants present several advantages when used to treat ADHD as they are fast acting, which results in improvements in ADHD symptoms in less than 1 h. However, the effects of stimulant medication are generally short-lived, often wearing off in less than 12 h.

Compared to stimulants, non-stimulants (e.g., atomoxetine, guanfacine, and clonidine) are a newer pharmacotherapy intervention to treat ADHD. Non-stimulants can be taken individually or in conjunction with stimulants to increase the effectiveness of treatment. In 2002, atomoxetine became the first non-stimulant medication approved by the FDA to treat ADHD (Hazell et al., 2011). Unlike the rapid effects of stimulants, atomoxetine, which is a selective noradrenaline reuptake inhibitor (SNRI), may take up to 4 weeks to observe a reduction in ADHD symptoms (Savill et al., 2015). Studies have shown that atomoxetine increases extracellular levels of dopamine and norepinephrine in the prefrontal cortex (Bymaster et al., 2002), thereby decreasing impulsivity and hyperactivity, as well as improving concentration. Studies have demonstrated atomoxetine efficacy in the long-term treatment of ADHD, but the study effect sizes have been smaller than for stimulants (Cortese et al., 2018).

Since the 1970's studies have rigorously investigated the effectiveness of medication in reducing symptoms of ADHD in children and adolescents. Most studies have utilized indirect assessment methods, primarily in the form of parent or teacher rating scales, and changes in performance on a variety of cognitive measures to evaluate the effects of medication on ADHD symptoms. However, more recently the neuropharmacological effects of medication on children and adolescents with ADHD have been conducted. These studies have largely been conducted using functional magnetic resonance imaging (fMRI). Studies of brain activity using fMRI have generally shown that for children and adolescents using stimulants there is a normalizing of brain activity during both resting state and task-based activities by way of increased activity in the bilateral inferior frontal cortex (Hart et al., 2013; Rubia et al., 2014) and improved suppression of activity in the default mode network (Peterson et al., 2009). Although few studies utilizing fMRI have been conducted on non-stimulant usage in children and adolescents, there is evidence that atomoxetine increases activation in the right inferior frontal gyrus (Chamberlain et al., 2009) and the right middle/superior temporal cortex, posterior cingulate, and precuneus regions (Kowalczyk et al., 2019). Thus, despite beneficial outcomes on ADHD symptomology for stimulant and non-stimulant medications, studies point to some differences in the neuropharmacological mechanisms associated with each medication (Cubillo et al., 2014a,b; Chou et al., 2015).

The advent of fMRI technology transformed the field of neuroimaging through images of brain metabolic function (Mier and Mier, 2015); however, using fMRI in the context of children and adolescents, and particularly those diagnosed with ADHD, may prove challenging. In particular, fMRI's high sensitivity to movement artifacts makes it a less than ideal option for children and adolescents with ADHD who tend to be hyperkinetic (Scarapicchia et al., 2017). In recent years, a novel, non-invasive neuroimaging technique - functional near infrared spectroscopy (fNIRS) – offers an alternative to fMRI. While fMRI offers superior spatial resolution and whole as fMRI and whole brain measurement, fNIRS has the advantage that it is less sensitive to movement artifact and may be portable, making it a useful tool for children and adolescents, including those diagnosed with ADHD.

fNIRS determines the changes in concentrations of oxygenated and deoxygenated hemoglobin in cortical regions using specific wavelengths (650 and 950 nm) of near-infrared light (Strangman et al., 2002). It can be used to measure brain activation in both resting and active states. Evidence that there is a linear relationship between hemodynamics and neural activity (Ma et al., 2016) allows fNIRS to reliably assess neural activation to a stimulus. Activation of brain structures in response to a stimulus results in increased blood flow and blood volume, which is determined by measuring local concentrations of oxyhemoglobin (HbO), deoxyhemoglobin (HbR), or the summed total (HbT) (Wilcox and Biondi, 2015). Despite having significantly weaker signal-to-noise ratio (SNR) than fMRIs and a lack of ability to detect activation in subcortical regions (Wilcox and Biondi, 2015), there is evidence showing that fNIRS measurements are consistent with those of fMRI and positron emission tomography (PET). Thus, fNIRS may be considered a reliable measure of brain activation and serves as a possible substitute for fMRI when considering children and adolescents with ADHD. Moreover, fNIRS may be a suitable neuroimaging option to evaluate the neuropharmacological effects of ADHD medications.

To date, fNIRS has been used in the context of children and adolescents with ADHD to primarily identify biomarkers of pharmacotherapy outcomes and assess the haemodynamic response to ADHD medication. In this systematic literature review we document and summarize the methodological and design variables of studies that have used fNIRS to investigate the cortical responses to drug administration and provide a brief synthesis of outcomes. Our aim was to synthesize the data from the articles that fit our inclusionary criteria to evaluate the different approaches to research in this field as well as make suggestions for future research based on a review of the results and limitations identified in these studies. We focused on documenting studies that were specific to children and adolescents with ADHD as the majority of this population is treated with medication. Our review was guided by the following questions:

- *What are the characteristics of the participants included?*
- *What designs were used to conduct these studies?*
- *What procedures and procedural variations were used to determine findings?*
- *What are the outcomes of a restricted sample of the studies from this review who adopted a similar research focus?*

## Methods

### Search strategy

Two of the authors (EP and MJO) conducted primary and reliability electronic searches, respectively. We utilized the Preferred Reporting Items for Systematic Reviews and Meta-Analyses (PRISMA) guidelines for our systematic literature review. We identified studies from PsychInfo, Scopus, and PubMed that included any of the multiple variations related to three main concepts: (1) fNIRS, (2) children and adolescents diagnosed with ADHD, and (3) medication. No limitations were set for publication year. The exact search strategy is included in Appendix 1.

### Inclusion and exclusion criteria

Inclusion and exclusion criteria were determined a priori. Studies were included if they were (1) empirical and (2) utilized fNIRS to (3) investigate the neural response of pharmacological agents targeting (4) ADHD in (5) children and adolescents (under age 18 years). Studies where participants had comorbid disorders such as autism spectrum disorder (ASD) and epilepsy were included. Studies that investigated the therapeutic effects of non-pharmacological intervention such as neurofeedback and transcranial electrical stimulation (TES), compared these treatments to medication, or were published in a language other than English, were excluded.

### Study selection

**Figure 1:**
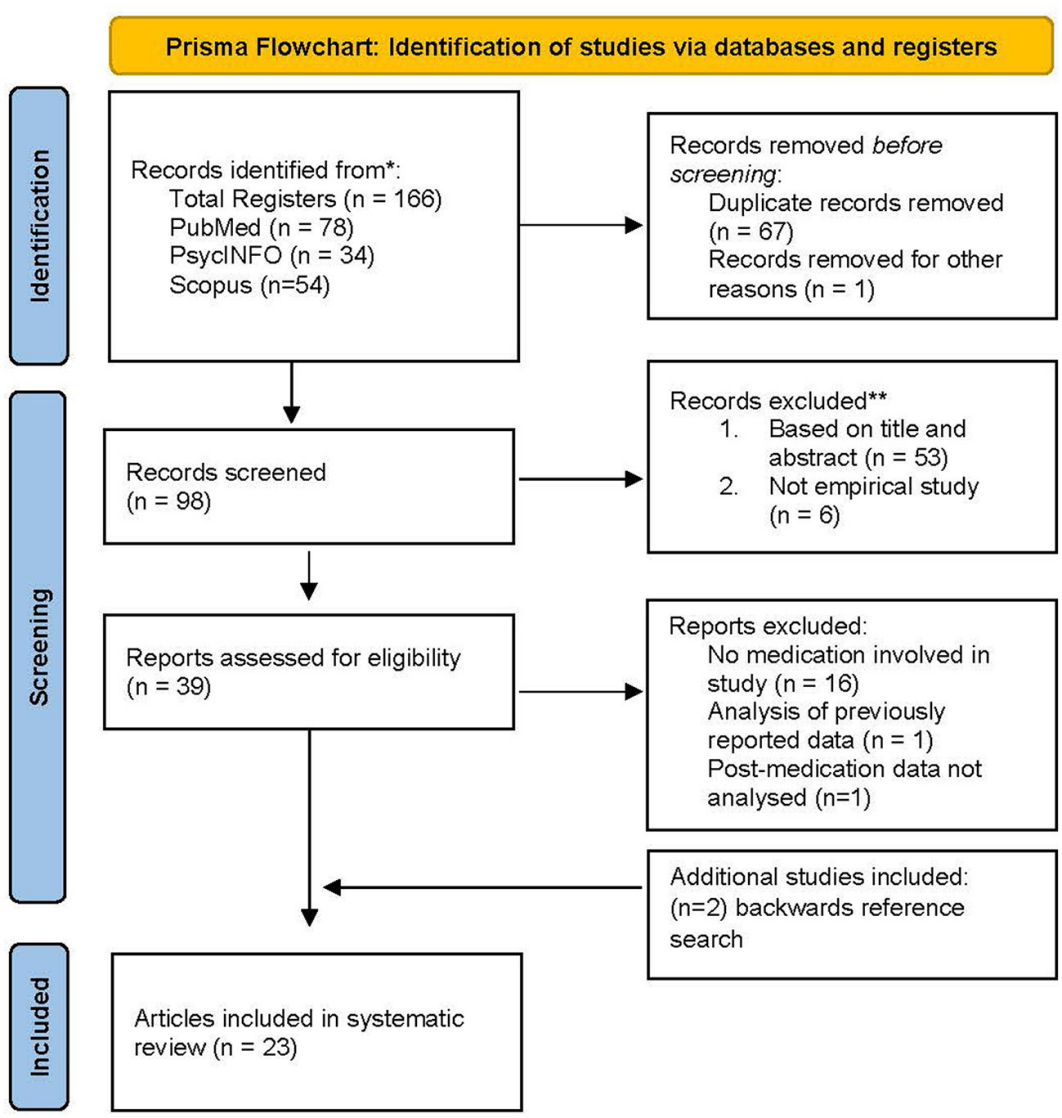
PRISMA flow diagram showing search and study selection.

The PRISMA flow diagram is shown in Figure 1. The search was conducted in July of 2022 and yielded 166 citations. After duplicate removal (67 records) and removal of one study not published in English, 98 citations remained. During screening, six records were excluded because they were not empirical studies, and 53 additional records were excluded following title and abstract screening. In total, 39 potentially eligible studies were identified. When the inclusion/exclusion criteria were applied, 16 additional studies were removed due to utilizing a non-pharmacological intervention or no intervention at all, one study was excluded because it used a previously published data set from another article included in this review, and one study was excluded as it did not analyze fNIRS data post medication. Two additional studies were identified through a backward reference search, and both met inclusion criteria. The final number of studies included in this review was 23. A reliability check for the initial search results yielded identical numbers to the initial search record. A two-step reliability check was then completed regarding the final number of eligible articles and verifying inclusion and exclusion decisions. Agreement was 100%.

## Results

### Participant demographics

Participant demographics are shown in Table 1. Across the 23 studies included in this review there were a total of 645 participants (177 typically developing individuals and 468 individuals diagnosed with ADHD, 32 of which served as medication naïve ADHD control participants). Studies in this review varied in the degree to which they described their participants, with many studies failing to report key demographic information. For example, < 53% of studies included data on participants' race and ethnicity, comorbid disorders, and cognitive abilities, and several included only the age and sample size. Participants varied moderately across several demographic characteristics. In terms of age, participants ranged from 6 to 16 years old. All but one study (Öner et al., 2011), provided data on participant gender, with females representing just 21.7% of all test participants (345 males to 75 females). Confirmation of participants' ADHD diagnosis was made using either the DSM-IV (15 studies) or DSM-5 (7 studies) diagnostic criteria, with the exception of one study (Dolu et al., 2019) that did not specify whether DSM criteria were used to confirm an ADHD diagnosis.

**Table 1:** Participant demographics from retrieved studies.

**References**	**No of participants with ADHD**	**Participant age in years (mean, range)**	**Gender: (male:female)**	**Control**	**Comorbidity**
Araki et al. (2015)	12	9.8, 6–13	6:6	14 – TD	ASD (*n =* 3); Anxiety disorder (*n =* 1)
Dolu et al. (2019)	18	9.55, 7–12	14:4	18 – TD	NR
Grazioli et al. (2021)	24	11, 6–16	22:2	25 – TD	SLD = 50%; ODD = 17%; Anxiety disorder = 13%; ASD = 8%; Mood disorder = 13%^*^
Ikeda et al. (2021)	12	8.2, 6–10	11:1	None	None
Ishii-Takahashi et al. (2015)	30	8.6, NR	26:4	20 – TD	NR
Jang et al. (2021)	23	9.96, 7–16	16:7	12 – TD	Tic disorder (*n =* 1)
Kawai et al. (2021)	10	9.3, 7–12	10:0	10 – TD	NR
Kobayashi et al. (2020)	19	9.84, 8–12	18:1	None	ASD (*n =* 6)
Li et al. (2022)	38	8.77, 6–12	29:9	None	None
Matsuura et al. (2014)	11	10.8, 10–15	10:1	None	None
Monden et al. (2012a)	12	9.7, 7–14	11:1	None	ASD (*n =* 2); Epilepsy (*n =* 1)
Monden et al. (2012b)	16	8.8, 6–13	10:6	16 – MN	ASD (*n =* 4); Epilepsy (*n =* 1)
Nakanishi et al. (2017)	30	NR, 6–14	MPH: 14:2 ATX: 11:3	None	None
Nagashima et al. (2014a)	16	8.9, 6–14	14:2	16 – MN	ASD (*n =* 13)
Nagashima et al. (2014b)	22	9.5, 6–14	19:3	22 – TD	ASD (*n =* 12); Epilepsy (*n =* 1)
Nagashima et al. (2014c)	15	NR, 6-14	12:3	15 – TD	ASD (*n =* 9)
Öner et al. (2011)	16	NR, 7–14	NR	None	Disorder not specified (*n =* 6)
Ota et al. (2015)	10	NR, 7-13	7:3	None	NR
Sanefuji et al. (2014)	7	10.4, 8–12	7:0	19 – TD	None
Schecklmann et al. (2011)	27	NR, 10–16	20:7	22 – TD	ODD (*n =* 10); Elimination disorder (*n =* 1); Specific phobia (*n =* 1)
Tokuda et al. (2018)	32	With ASD: 8.2, 7–14 Without ASD: 7.8, 6–13	With ASD: 11:0 Without ASD: 17:4	None	ASD (*n =* 11)
Weber et al. (2007)	10	10.7 (median), 8–11	10:0	None	NR
Wigal et al. (2012)	26	NR, 6–12	20:6	None	NR

NR, not reported; TD, typically developing (no ADHD diagnosis); ASD, autism spectrum disorder; ODD, oppositional defiant disorder; MPH, methylphenidate; ATX, atomoxetine. MN, medication naïve (diagnosed with ADHD).

* Based on entire ADHD sample, of which 9 participants were not included in the fNIRS data for various reasons.

Roughly half of the included studies (56.5%) specified the ADHD presentation or subtype of participants. Across these 13 studies, combined presentation was reported most frequently, representing 74% of participants. This was followed by primarily inattentive presentation (24%), and primarily hyperactive and impulsive presentation (2%). Among those studies reporting presentations, two (Öner et al., 2011; Sanefuji et al., 2014) only included participants with combined presentation, while all others included participants with any presentation.

Several studies included participants with comorbid diagnoses, but not all studies made explicit whether there were comorbid diagnoses or what those diagnoses were. Twelve studies (52.2%) included participants with comorbid diagnoses and five studies (21.7%) excluded individuals with a comorbid diagnosis. More than 20% of all studies (5 total) did not provide any information on whether participants had a comorbid diagnosis and if so, what those diagnoses were. One study (Ishii-Takahashi et al., 2015) allowed for participants with a comorbid disorder to participate if the primary diagnosis was ADHD, but the study did not make mention of the number of participants with a comorbid diagnosis. Among the studies reporting a comorbid diagnosis, ASD was the most common. Other comorbid diagnoses included: epilepsy, anxiety, conduct disorder, oppositional defiant disorder, specific learning disorder, specific phobias, elimination disorder, tic disorder, and depression.

Studies tended to be relatively small, with a mean of 19 test (non-control) participants (range, 7 to 36). More than half of the studies (13 studies) included 16 or fewer participants and just more than 17% (4 studies) included 30 or more participants. In 11 studies (46%), participants served as their own controls (i.e., on and off medication). Among the 12 studies that used a control group, the majority (83%) used typically developing children and two (Monden et al., 2012b; Nagashima et al., 2014a) used non-medicated individuals with ADHD as controls. All studies utilizing a control group matched the samples for gender and age, except Schecklmann et al. (2011), in which the control and ADHD groups did not differ in age but differed significantly in gender distribution, and thus gender was introduced as a covariate.

### Pharmacological variables and procedures

#### Types of medications

As shown in Table 2, across the 23 studies, three classes of medication were evaluated. The majority of studies (67%) looked solely at the effects of stimulants, while a smaller number of studies (17%) evaluated atomoxetine and one study evaluated an extended-release antihypertensive medication (i.e., guanfacine extended release). Additionally, one study included both methylphenidate and atomoxetine in their analysis. Of the 18 studies that evaluated a stimulant medication, immediate-release methylphenidate was included in 17 studies and the remaining study included extended-release lisdexamfetamine.

**Table 2:** Procedural variables from retrieved studies.

**References**	**Med**	**Med naïve**	**Range of exposure length**	**Washout period**	**Task**
Araki et al. (2015)	ATX	Yes	NR	No	CPT - visual stimuli
Dolu et al. (2019)	MPH	Yes	NR	NR	Oddball
Grazioli et al. (2021)	MPH	Yes	N/A	No	e-CPT
Ikeda et al. (2021)	GXR	No	8–24 months	4 days	Go/No-Go
Ishii-Takahashi et al. (2015)	MPH	Mix	>1 month	1 week	Stop signal
Jang et al. (2021)	MPH	No	NR	2 days	N-back
Kawai et al. (2021)	MPH	NR	NR	2 weeks	Stroop
Kobayashi et al. (2020)	MPH	No	0.2–3.4 months	4 days	Facial emotion recognition task
Li et al. (2022)	MPH	Yes	N/A	N/A	Go/No-Go
Matsuura et al. (2014)	MPH	NR	NR	24 h	CANTAB executive function tasks
Monden et al. (2012a)	MPH	Mix	1 week−3.6 years	>24 h	Go/No-Go
Monden et al. (2012b)	MPH	Mix	0.1–3.4 years	4 days	Go/No-Go
Nagashima et al. (2014a)	ATX	No	2–27 months	2 days	Go/No-Go
Nagashima et al. (2014b)	MPH	No	1–61 months.	4 days	Oddball
Nagashima et al. (2014c)	ATX	No	2–41 months	2 days	Oddball
Nakanishi et al. (2017)	MPH (*n* = 16) ATX (*n* = 14)	Yes	N/A	No	Stroop
Öner et al. (2011)	MPH	Yes	N/A	N/A	Stroop
Ota et al. (2015)	ATX	Yes	N/A	No	Stroop
Sanefuji et al. (2014)	MPH	Mix	NR	>24 h	Short term memory
Schecklmann et al. (2011)	MPH	No	Group 1 = 39.5 ± 17.1 months; Group 2 = 30.6 ± 18.6 months	>14 half-lives of MPH	Olfactory
Tokuda et al. (2018)	MPH	Yes	N/A	N/A	Go/No-Go
Weber et al. (2007)	MPH	Yes	N/A	N/A	Trail making
Wigal et al. (2012)	LDX	NR	NR	2 weeks	None

NR, not reported; N/A, not applicable MPH, methylphenidate; ATX, atomoxetine; GXR, guanfacine; LDX, lisdexamfetamine.

#### Approach

Studies generally took one of three approaches to evaluate medications. Sixty-one percent of studies (14 studies) focused solely on the immediate effect of medication (i.e., fNIRS measurement after a single administration, usually 1 to 4 h post administration), while 26.1% (6 studies) evaluated the effects of medication over a period of time (i.e., fNIRS measurement at multiple time points, often more than 1 month from baseline). An additional three studies considered both the immediate effects and the long term effects of medication: Li et al. (2022) considered the immediate effects (1.5 h following administration) and long-term effects (following 4 weeks of administration) of methylphenidate; Ishii-Takahashi et al. (2015) also evaluated both the immediate and long-term therapeutic effects of methylphenidate and took fNIRS measurements 5 h after a single dose of methylphenidate, after 4–8 weeks of continuous methylphenidate administration, and then 1 year after continuous methylphenidate administration; Wigal et al. (2012) looked at the effects of lisdexamfetamine 2–4 h after intake and then 3–4 weeks from first medication exposure.

Among the studies evaluating the immediate effects of ADHD medication, fNIRS measurements were conducted in one of three different schedules. In total of 42.9% took a single fNIRS measurement across two different visits (up to 1 month apart) with participants either off medication/on placebo for the first visit and on medication for the second visit, or vice versa, which allowed for pre/post comparisons. Similarly, 42.9% took fNIRS measurements twice (once on medication and once off medication) for each of two visits (up to 1 month apart), which allowed for same day on-/off-medication analysis at two timepoints. Finally, two studies (Monden et al., 2012a; Jang et al., 2021) also took two fNIRS measurements on the same day, but only for a single visit (once on medication and once off medication).

All six studies that looked at the long-term response to medication took two fNIRS measurements at intervals ranging from 1 month (Grazioli et al., 2021) up to a maximum of 1 year (Araki et al., 2015), while studies exploring both the immediate- and long-term effects of medication took three or more measurements. Wigal et al. (2012) and Li et al. (2022) conducted fNIRS measurements three times and Ishii-Takahashi et al. (2015) conducted between four and five fNIRS measurements depending on whether participants were or were not medication-naïve at the start of the study.

### Immediate response studies (14 studies)

Studies considering the immediate neural response of medication were focused largely on methylphenidate (11 out of 14 or 78.6%); however, two studies included atomoxetine (Nagashima et al., 2014a,c) and one study (Ikeda et al., 2021) evaluated extended release guanfacine. Half of all immediate-response studies included only participants with previous exposure (i.e., current users) to the medication being investigated, whereas 21.4% used a combination of medication-naïve participants and medication exposed participants, and 14.3% of the studies used only medication-naïve participants. The rest of the studies (14.3%) did not report whether participants were medication naïve.

When participants with previous medication exposure were included (11 studies total), a washout period was always implemented to ensure no carryover effects were observed. This period was between 24 h and 2 weeks for methylphenidate, 2 days for atomoxetine, and 4 days for extended release guanfacine.

With regards to dosage for studies evaluating methylphenidate (11 studies), 18.2% used a fixed dosage, ranging from 10 mg to 54 mg, and administration occurred between 45 min and 4 h preceding fNIRS tests for immediate-release methylphenidate and up to 5 h for extended-release methylphenidate. When participants were methylphenidate exposed prior to participation, exposure ranged from 0.2 to 61 months. For non-stimulant studies, the dosage of atomoxetine ranged from 5 to 50 mg, whereas the dosage of guanfacine was fixed at 1 mg/day. With regards to timing, both atomoxetine studies took fNIRS measurements 1.5 h post medication intake. Two studies (Schecklmann et al., 2011; Kawai et al., 2021) did not report the medication dosage.

### Long-term response studies (6 studies)

One third of the studies evaluating the long-term neural response to medication focused on atomoxetine, which is reasonable given that atomoxetine often takes more than 1 month to reach optimal therapeutic effects (Clemow and Bushe, 2015). Three of six studies investigated the long-term response using methylphenidate. Additionally, one study (Nakanishi et al., 2017) investigated both atomoxetine and methylphenidate.

All long-term response studies solely evaluated individuals with no previous ADHD medication exposure (medication-naïve). Studies either utilized a standard timing for medication administration of 1.5 h prior to fNIRS measurement or did not state how long before the fNIRS measurement medication was administered. Due to the focus on long-term response to medication, there was greater variability in the way medication was administered across studies. Four studies titrated the dosage across time and defined a terminal dosage stopping point. Araki et al. (2015) titrated until ADHD symptoms clinically improved; Dolu et al. (2019) titrated until “optimal dosage”, although the definition for this was unclear; Weber et al. (2007) titrated until a target dose of 10 mg twice daily was reached; and Nakanishi et al. (2017), which looked at both atomoxetine and methylphenidate, titrated dosage until participants reached the lowest effective dose (mean dose of methylphenidate was 0.87 mg/kg and mean dose of atomoxetine was 1.30 mg/kg). For studies evaluating atomoxetine only, average dosage was 1.6 mg/kg/day (Araki et al., 2015) and 1.34 mg/kg (Ota et al., 2015), while the average dosage in the two studies evaluating methylphenidate only were 0.33 mg/kg/dose (median; Weber et al., 2007) and 41.14 mg/g (mean; Dolu et al., 2019).

One study Grazioli et al. (2021) did not report the specific dosages and stated that doses were adjusted based on treatment response and tolerability and were maintained until the second evaluation of the study after the second week.

### Mixed procedure studies (3 studies)

A total of three studies looked at a mixture of the immediate and long-term neural response of medication. Two studies investigated methylphenidate, and one study (Wigal et al., 2012) evaluated lisdexamfetamine. Studies by Wigal et al. (2012) and Li et al. (2022) took fNIRS measurements at three time points: baseline, immediate response (1.5 to 4 h) following first administration and immediate response following 4 weeks of continuous medication administration. Ishii-Takahashi et al. (2015) conducted fNIRS measurements at four time points for non-naïve medication participants and at five time points for medication naïve participants. Both non-naïve and naïve participants had fNIRS measurements at baseline, immediately following a single dose of methylphenidate, after 4 to 8 weeks of continuous medication administration. Medication-naïve participants had an additional fNIRS measurement taken at 1 year follow up. Ishii-Takahashi et al. (2015) was the only study of the three to use a mixture of medication-naïve and non-naïve participants (at least 1 month of exposure to methylphenidate prior to study), with a washout period of 1 week prior to baseline assessment.

Across the three studies conducting a mixed procedure, only one study (Li et al., 2022) used a fixed dose (18 mg methylphenidate). Ishii-Takahashi et al. (2015) utilized a fixed dose of methylphenidate (18 mg) when looking at the initial immediate response to methylphenidate, and then titrated until optimal dosage (*M* = 25.4 mg). Wigal et al. (2012) used a fixed dosage of lisdexamfetamine at each of the first three visits (30, 50, and 70 mg respectively) and then determined the optimal dosage based on participant response after the third visit.

### fNIRS equipment and measurement

As shown in Table 3, the majority of studies in this review (56.5%) utilized fNIRS devices manufactured by Hitachi Medical Corp and, with one exception, the device was the ETG-4000 model. Among the rest of the studies, 11 different models from seven different manufacturers were used. All devices were designed as continuous wave measurement systems and used a clinically safe light wavelength, ranging from 690 to 850 nm. The number of channels used ranged from 2 (Weber et al., 2007; Wigal et al., 2012) to 48 (Jang et al., 2021), with a mode of 22 channels (7 of 23 studies). Most studies used an emitter-detector distance of 3 cm (74%). Sampling rates ranged from 1.7 to 15.625 Hz. Oxygenated Hb was most frequently the single measure of interest (65%). A variety of filtering methods were used for noise (e.g., motion, heart, and respiration) removal, but details on motion correction and baseline correction were not reported for the majority of studies (74 and 65%, respectively).

**Table 3:** fNIRS set-up and processing from retrieved studies.

**References**	**fNIRS MFG**	**fNIRS model**	**No of channels**	**Emitter-detector distance**	**Sampling rate**	**Measures of interest**	**Noise removal**	**Motion correction**	**Baseline correction**	**Region of interest**
Araki et al. (2015)	Hitachi Medical Corp.	ETG-100	24	3 cm	10 Hz	Oxy-Hb, Deoxy-Hb	NR	Moving average (10 s)	Linear fitting based on pre-task (6 s before task onset) and post-task (mean across a 6 s period, 12 s after task period)	Frontal cortex: Superior frontal Middle frontal Inferior frontal
Dolu et al. (2019)	NR	NR	16	NR	2 Hz	Oxy-Hb	Low-pass filtered (cut-off of 0.14 Hz)	NR	NR	Frontal cortex
Grazioli et al. (2021)	NIRx Medical Technologies	DYNOT Compact 9–32	14	3 cm	NR	Oxy-Hb	Third order Butterworth low-pass filter (cut-off of 1 Hz)	Moving average (5 s) Wavelet-based correction	NR	Frontal cortex Pre-frontal cortex
Ikeda et al. (2021)	Hitachi Medical Corp.	ETG-4000	22	3 cm	10 Hz	Oxy-Hb	First degree polynomial fitting High-pass filter (0.01 Hz) for baseline drift Low-pass filter (0.8 Hz)	NR	NR	Frontal cortex: DLPFC LPFC Parietal cortex Temporal cortex
Ishii-Takahashi et al. (2015)	Hitachi Medical Corp.	ETG-4000	24	3 cm	10 Hz	Oxy-Hb	NR	Moving average (5 s)	Linear fitting based on pre-task (10s before task onset) and post-task (mean across last 10 s after task period)	Pre-frontal cortex: IPFC
Jang et al. (2021)	OBELAB	NIRSIT	48	3 cm	8.138 Hz	Oxy-Hb	Band-pass filter (0.005–0.01)	NR	NR	Pre-frontal cortex: DLPFC VLPFC MPFC OFC
Kawai et al. (2021)	Shimadzu Corp.	NIRStation - OMM 3000	17	NR	NR	Oxy-Hb	NR	NR	Set to zero at start of stimulus Smoothing *via* Savitzky-Golay method	Pre-frontal cortex: DLPFC
Kobayashi et al. (2020)	Hitachi Medical Corp.	ETG-4000	44	3 cm	10 Hz	Oxy-Hb, Deoxy-Hb	First degree polynomial fitting High-pass filter (0.01 Hz) for baseline drift Low-pass filter (0.8 Hz)	NR	NR	Temporal cortex
Li et al. (2022)	NIRx Medical Technologies	NIRSport	16	3 cm	15.625 Hz	Oxy-Hb, Deoxy-Hb	“Pre-processed to eliminate discontinuities and remove spikes from head movements” High-pass filter (0.01 Hz) and low-pass filter (0.8 Hz)	NR	NR	Pre-frontal cortex
Matsuura et al. (2014)	Spectratech	OEG-16	16	3 cm	NR	Oxy-Hb	NR	NR	Linear fitting based on pre-task (10s before task onset) and post-task (mean across last 10 s after task period)	Pre-frontal cortex
Monden et al. (2012a)	Hitachi Medical Corp.	ETG-4000	22	3 cm	100 ms	Oxy-Hb, Deoxy-Hb, Total Hb	First degree polynomial fitting High-pass filter (0.01 Hz) for baseline drift Low-pass filter (0.8 Hz)	NR	NR	Pre-frontal cortex: DLPFC VLPFC
Monden et al. (2012b)	Hitachi Medical Corp.	ETG-4000	22	NR	NR	Oxy-Hb	First degree polynomial fitting High-pass filter (0.01 Hz) for baseline drift Low-pass filter (0.8 Hz)	NR	NR	Pre-frontal cortex: LPFC
Nagashima et al. (2014a)	Hitachi Medical Corp.	ETG-4000	22	3 cm	NR	Oxy-Hb	First degree polynomial fitting High-pass filter (0.01 Hz) for baseline drift Low-pass filter (0.8 Hz)	NR	NR	Pre-frontal cortex: LPFC Parietal cortex: Inferior
Nagashima et al. (2014b)	Hitachi Medical Corp.	ETG-4000	22	3 cm	NR	Oxy-Hb	First degree polynomial fitting High-pass filter (0.01 Hz) for baseline drift Low-pass filter (0.8 Hz)	NR	NR	Pre-frontal cortex: LPFC Parietal cortex: Inferior
Nagashima et al. (2014c)	Hitachi Medical Corp.	ETG-4000	22	3 cm	NR	Oxy-Hb	First degree polynomial fitting High-pass filter (0.01 Hz) for baseline drift Low-pass filter (0.8 Hz)	NR	NR	Pre-frontal cortex: LPFC parietal cortex: inferior
Nakanishi et al. (2017)	Hitachi Medical Corp.	ETG-4000	24	3 cm	NR	Oxy-Hb	NR	Moving average (5 s)	Integral Mode: Linear fitting based on pre-task (10 s before task onset) and post-task (25 s after task period)	Cerebral cortex
Öner et al. (2011)	NR	NIROXCOPE 301	16	2.5 cm	1.7 Hz	Oxy-Hb, Deoxy-Hb	Low pass filter (0.33 Hz)	NR	NR	Pre-frontal cortex: DLPFC
Ota et al. (2015)	Hitachi Medical Corp.	ETG-4000	24	3 cm	NR	Oxy-Hb	NR	Moving average (5 s)	Linear fitting based on pre-task (10 s before task onset) and post-task (25 s after task period)	Frontal cortex
Sanefuji et al. (2014)	Shimadzu Corp.	OMM-2001	6	3 cm	70 ms	Total Hb	NR	NR	NR	Parietal cortex: DLPFC VLPFC
Schecklmann et al. (2011)	Hitachi Medical Corp.	ETG-4000	24	3 cm	10 Hz	Oxy-Hb	NR	Moving average (5 s)	Linear fitting based on pre-task (10 s before task onset) and post-task (10–20 s after task period)	Frontal cortex: Inferior Temporal cortex
Tokuda et al. (2018)	Hitachi Medical Corp.	ETG-4000	22	3 cm	10 Hz	Oxy-Hb	First degree polynomial fitting High-pass filter (0.01 Hz) for baseline drift Low-pass filter (0.8 Hz)	NR	NR	Frontal cortex: inferior middle
Weber et al. (2007)	Hamamatsu Photonics KK	NIRO-300 spectrometer	2	NR	2 Hz	Oxy-Hb, Deoxy-Hb, Total Hb, tissue oxygenation index	NR	NR	NR	Parietal cortex: APFC DLPFC
Wigal et al. (2012)	ISS	Oximeter	2	2–3.56 cm	9.75 Hz	Oxy-Hb, Deoxy-Hb	NR	NR	NR	Frontal cortex

NR, not reported; DLPFC, dorsolateral pre-frontal cortex; LPFC, lateral pre-frontal cortex; IPFC, inferior pre-frontal cortex; VLPFC, ventrolateral pre-frontal cortex; MPFC, medial pre-frontal cortex; OFC, orbitofrontal cortex; APFC, anterior pre-frontal cortex.

Although studies evaluated multiple brain regions of interest, the frontal lobe was targeted in more studies than any other region (70%). For studies looking at the frontal lobe, three studies had a general focus on the frontal lobe, while the other 13 specified measurement within the prefrontal cortex. Several studies explored other lobes, including four that targeted the parietal cortex and one that evaluated the parietal and temporal cortex. Two studies focused only on the temporal cortex. All studies evaluated effects bilaterally.

Studies conducted fNIRS measurements during a variety of tasks (see Table 2), with one study (Wigal et al., 2012) only conducting fNIRS measurements in resting state. Studies nearly exclusively utilized executive functioning tasks (87%), with the two most common being a go/no go task (6 out of 20 studies or 30%) and the Stroop test (3 studies or 15%). Eight other executive functioning tasks were used across the remaining studies. Of the two studies that did not use an executive functioning task, one utilized an olfactory sensitivity task (Schecklmann et al., 2011) and the other (Kobayashi et al., 2020) employed an affect recognition task.

### fNIRS outcomes

#### Baseline outcomes

As noted previously, the majority of studies (*n* = 18; 75%) used (a) executive functioning tasks to (b) assess brain activation on and/or off medication. To ensure a high level of comparability across studies, we restricted our review of results to those 18 studies.

Only 11 studies reported pre-medication outcomes; all found under activation or no expected increase in activation in the region targeted during the tasks, but the details reported varied. Nine studies specified the laterality of the under activation, with the right hemisphere being the most common. Within the right hemisphere, three studies showed under activation in the general pre-frontal region, while other studies demonstrated under activation in more specific regions, including the right inferior frontal gyrus/middle frontal gyrus (2 studies), dorsolateral pre-frontal cortex and the left ventrolateral pre-frontal cortex (2 studies), inferior parietal lobe (1 study), and inferior frontal cortex (1 study). Overall, at the baseline (pre-medication) level, there was relative consistency across studies reporting outcomes, with evidence suggesting lower activation of the regions of interest.

### Treatment outcomes

#### Methylphenidate

Findings were variable across studies that focused on the effects of methylphenidate on the frontal region (*n* = 12). Seven studies saw an increase in neural activity. Among those seven studies, five observed the increase in the pre-frontal cortex and four more identified increased activation specifically in the right pre-frontal cortex. One study (Ishii-Takahashi et al., 2015) observed an increase in the right inferior frontal cortex, and another study (Monden et al., 2012b) observed increased activation in the right inferior frontal gyrus/middle frontal gyrus.

Additional methylphenidate studies found increased activation in targeted regions, but the findings were more nuanced. For example, Matsuura et al. (2014) observed increased activation in the pre-frontal cortex, but only on more complex executive functioning tasks that focused on visuospatial working memory. Studies that compared fNIRS results using groups defined by genotype also displayed mixed findings. Li et al. (2022) demonstrated increased activation in the dorsolateral pre-frontal cortex after 4 weeks of methylphenidate for participants with a T/T genotype, but no difference in brain activation was observed in the same region over the same time period for participants in the G allele carrier group. Additionally, Öner et al. (2011) evaluated the immediate response to methylphenidate with a mixture of adult and child participants with SNAP-25 polymorphism and found significant differences in dorsolateral pre-frontal cortex activation between genotype subgroups.

Only one study (Grazioli et al., 2021) did not find increased activation in the targeted brain regions post medication. In this study, Grazioli et al. (2021) failed to demonstrate brain activation in the pre-frontal region following methylphenidate administration and they reasoned that fNIRS may not have been sensitive enough to identify changes that may have occurred subcortically.

In the final study (Jang et al., 2021), decreased activation was observed in both the dorsolateral pre-frontal cortex and the medial pre-frontal cortex. This study represented the only study using methylphenidate that showed decreased activation in a targeted brain region.

#### Atomoxetine

In studies where fNIRS measured brain activity responding to atomoxetine during executive functioning tasks (*n* = 4), consistent increases in activation were demonstrated. Decreased activation in multiple brain regions (left ventrolateral pre-frontal cortex, right dorsolateral pre-frontal cortex, right inferior frontal gyrus/middle frontal gyrus, and inferior parietal lobe) observed during baseline were normalized following atomoxetine administration. The one exception came from Araki et al. (2015) who found normalized activation in the right dorsolateral pre-frontal cortex, but not in the left ventrolateral pre-frontal cortex.

#### Methylphenidate and atomoxetine

In the study that investigated the effects of both methylphenidate and atomoxetine (Nakanishi et al., 2017), increased activation was observed in the pre-frontal cortex post-atomoxetine administration, but no significant changes were seen post methylphenidate intake.

#### Guanfacine

The single study to evaluate extended release guanfacine (Ikeda et al., 2021) failed to show any changes in the regions of interest (i.e., lateral pre-frontal cortices and parts of the frontal, parietal, and temporal lobes); however, an unexpected increase in activation was demonstrated in the right angular gyrus post medication.

## Discussion

This review of studies utilizing fNIRS to examine medication effects on neuronal activity in children and adolescents with ADHD revealed several important findings that contribute to the understanding of a promising approach to evaluating medication effects and highlight areas for future fNIRS researchers to consider when continuing to advance the medication efficacy science. Although our review considered a broad range of study variables, a major finding is that among the 18 studies for which we considered the outcomes, the results were highly consistent. Most demonstrated increased oxygenated hemoglobin concentrations in the pre-frontal cortex following pharmacotherapy. Additionally, the convergent findings from this review are highly consistent with outcomes from fMRI research (e.g., Rubia et al., 2014).

Despite consistent outcomes for a select number of studies, an equally important finding is that across all 23 studies included in this review, there was a high degree of methodological heterogeneity in terms of study demographics, medication considerations and brain regions of interest. Understandably, different approaches and study designs are likely when the aims of the studies vary as well. Nonetheless, in order to synthesize study results and come to more definite conclusions, some consistency is important, especially given the small sample size across studies (i.e., only 17.4% of studies used a sample size of 30 or more participants). Further, it is important to acknowledge the potential impact that the variables considered in this review may have on study results and the implications when designing future experiments that employ fNIRS to investigate the effects of medications in children and adolescents with ADHD. Relatedly, a significant issue that we identified across the studies was the inconsistency in the reporting of study details related to the experimental procedures, especially for participant demographics and medication administration procedures. This creates both a challenge in synthesizing the research literature and any attempt to replicate a study's findings.

### Study demographics

The subjects in the reviewed studies were all diagnosed with ADHD; however, ADHD itself is a heterogenous population and relatively few studies considered the possibility that neurobiological differences between individuals with different ADHD presentations may lead to different results. In fact, research has revealed subtle differences in neural connectivity between individuals with ADHD combined presentation and individuals with ADHD inattentive presentation (Saad et al., 2020) and if these differences are not accounted for in sample selection the results may be questioned. Only two studies (Öner et al., 2011; Sanefuji et al., 2014) restricted their samples to individuals with a common ADHD presentation (combined presentation for both) and many either did not collect information on ADHD presentation or did not report it. Future studies should specify the subtypes for all participants in their samples and consider analyses that would distinguish whether there are differences in the haemodynamic response to ADHD medication between ADHD subtypes.

In addition to ADHD presentation type, neurobiological differences are likely with various co-occurring diagnoses. A substantial degree of variability was observed in the inclusion/exclusion criteria for the studies in this review, and with regards to co-occurring conditions, only a fraction of the studies made clear that participants with specific co-occurring conditions were excluded. More problematic is that among the studies that did include individuals with co-occurring conditions some omitted details on the co-occurring conditions and/or numbers of participants with such conditions. This omission of details makes it difficult to determine whether medication effects vary as a result of comorbid conditions. Comorbidity is common in individuals with ADHD (Thapar and Cooper, 2016) and research has identified neurobiological differences between individuals only diagnosed with ADHD and those with ADHD and a co-occurring condition (Proal et al., 2013; Chantiluke et al., 2014). This underscores the need for studies like those included in this review to fully report and evaluate the possible differences in medication effects for individuals with and without co-occurring conditions.

### Medication considerations

The approaches in the reviewed studies could generally be broken down into those interested in the immediate effects (i.e., single use), those interested in the long-term effects (i.e., use over 1 month or greater) and those interested in comparing the immediate and cumulative (long-term) effects of medication on brain activation. The latter two approaches are better suited to studies of non-stimulants, which often take much longer to reach a therapeutic effect (Harpin, 2008). Regardless of the medication targeted, each of these research questions requires consideration of numerous factors, such as appropriate washout periods, optimal medication dosage, allowance for polypharmacy and current or previous target medication exposure, and the length of exposure prior to study procedures (for those using medication non-naïve participants). Not surprisingly, there was considerable variability across these factors and an unfortunate omission of specific details related to each for many studies.

Of particular importance is the variation in dosage used for the medications of interest. For methylphenidate, clinical practice guidelines (e.g., Wolraich et al., 2019) generally recommend dose “optimization” by starting with a low dose and titrating upwards until the maximal benefits are achieved with minimal adverse effects. Several studies in this review – all of which were interested in the long-term effects of medication – used this approach (Weber et al., 2007; Nakanishi et al., 2017; Dolu et al., 2019), which bolsters the external validity of their findings. However, what these studies gain in external validity they risk losing in internal validity, an advantage that studies using a fixed dose regimen gain. Studies that looked at the immediate effects of medication were more likely to use a fixed dosage, but the rationale for the selected dosage was often absent. Additionally, a few studies, including those interested in the immediate effects as well as those interested in the long-term effects of stimulants, neither titrated the dosage of methylphenidate nor used a fixed dosage. In these studies, only the mean dosage and range were reported without any indication of how the dosages were chosen. Ultimately, while there may not be merely one proper approach to dosing in studies examining medication efficacy using fNIRS, the large heterogeneity in approach to dosage does make it difficult to synthesize results and replicate findings. Future studies should make clear the benefits and limitations of their approach, the rationale for the chosen approach, and in the case of fixed or variable dosage studies (without titration) the rationale for the selected dose(s).

Whether the study was focused on the immediate or long-term impact of medication on brain activation, consideration for participant medication history, particularly with regards to the targeted medication, was inconsistent across studies. Several studies failed to report whether participants were medication naïve or non-medication naïve before the start of the study, and among the studies that included a mixture of medication-naïve and non-medication naïve participants, there was no indication of whether differences in brain activation between these two groups was identified prior to medication administration. Consideration for pre-administration differences is important as Ishii-Takahashi et al. (2015) demonstrated that differences in brain activation were exhibited between medication naïve and medication non-naïve participants pre-medication administration, with significantly lower pre-frontal activation observed in the naïve group. This shows that valid conclusions about the effects of ADHD medication may only be formed if differences in brain activation between medication naïve and medication non-naïve participants are accounted for during data analysis of fNIRS results. Furthermore, the Ishii-Takahashi et al. (2015) study emphasizes the importance of specifying the medication status of participants at the start of the study and reporting any differences in brain activation observed between medication-naïve and medication non-naïve participants when the sample includes a mixture of participants.

### Brain regions of interest

Finally, there was some inconsistency across the studies related to specificity of the region of interest targeted for assessment. While the majority of studies targeted the frontal cortex, only half provided more detailed information on activation within specific regions of the pre-frontal cortex (e.g., medial, orbital, dorsolateral). This limits the interpretation of results and may lead to overgeneralization. However, narrowly targeting a sub-region introduces the potential that changes in brain activation may be missed in other sub-regions not considered in the study. In fact, two studies (Nakanishi et al., 2017; Grazioli et al., 2021) that failed to observe changes in brain activity following medication administration suggested that their lack of positive finding may have been due to methylphenidate increasing activation in brain sub-regions that were not investigated with fNIRS.

## Conclusion and suggestions for future research

Despite its relatively recent genesis as a neuroimaging modality, fNIRS has emerged as an important tool for current and future research across a variety of fields, including neuroscience, psychology, psychiatry, and education and it has already begun to have an impact on the study of a variety of neurodevelopmental disorders. For example, resting state and task-based paradigms have been used to evaluate activation differences among young children who are and are not high risk for ASD, with the clinical goal of identifying early quantitative biomarkers (Conti et al., 2022). Further, fNIRS has been used to evaluate differences in brain activation during physical movement between children with and without cerebral palsy (CP; Sukal-Moulton et al., 2014) and to explore activation changes during treatment with the goal of intervention individualization (Cao et al., 2015; Perpetuini et al., 2022). Although fNIRS may still be considered a novel neuroimaging tool in comparison to other modalities (e.g., electroencephalogram, fMRI), outcomes of fNIRS studies with ADHD participants have demonstrated convergence with fMRI findings, supporting the clinical usefulness and reliability of fNIRS as a tool in ADHD research (Gossé et al., 2022). Research using fNIRS in ADHD has already been expansive. Studies have included characterization of the disease through comparisons of brain activity between individuals with and without an ADHD diagnosis (Monden et al., 2015), comparisons across different ADHD subtypes or presentations (Altinkaynak et al., 2017), and by isolating different forms of executive dysfunction (Hou et al., 2022; dos Santos Afonso et al., 2023). Additionally, fNIRS research has contributed to the exploration of potential early biomarkers among toddlers at high risk for ADHD (Kerr-German et al., 2022) and evaluation of treatment, including use of neurofeedback as a potential intervention (Kohl et al., 2020; Wu et al., 2022).

The extension of fNIRS to the evaluation of pharmacological treatment in children and adolescents with ADHD is promising. Although a great deal of research has been done using behavioral measures to identify optimal dosage of medication, fNIRS may be a viable neuroimaging tool to further study the neural changes that underly the behavioral changes associated with medication. Moreover, research has begun to investigate whether fNIRS can be utilized as tool to help select the optimal dosage of medication for children and adolescents with ADHD, with or in lieu of behavioral measures. Future research should expand into protocols and study structures that will take advantage of the unique capabilities that fNIRS offers. This might include expanding research to younger participant samples in an effort to evaluate developmental changes associated with ADHD and potential biomarkers, measuring neural activation in more naturalistic settings with more naturalistic tasks (e.g., completing schoolwork within a classroom while encountering different types of distractors), and taking advantage of the portability offered by some models to reach populations who may be less likely to travel to a lab for assessment. This expansion to protocols possible outside of a scanner will hopefully increase the generalizability and clinical utility of findings, including those focused on the effects of medication.

## Data availability statement

The original contributions presented in the study are included in the article/Supplementary material, further inquiries can be directed to the corresponding author.

## Author contributions

EP, AC, KS, and MO'B contributed to the study conception and design of the systematic literature review. EP and MO'B contributed to the electronic data search, study eligibility screening, data extraction, data analysis, and drafted the manuscript. AC and KS reviewed the manuscript and assisted with editing and revisions. All authors approved of the final version of the manuscript.
