# Supplementary figures and images for: Using fNIRS to evaluate ADHD medication effects on neuronal activity: A systematic literature review
Source: Front Neuroimaging. 2023 Jan 24;2:1083036. doi: 10.3389/fnimg.2023.1083036 (PMC10078617; doi:10.3389/fnimg.2023.1083036)

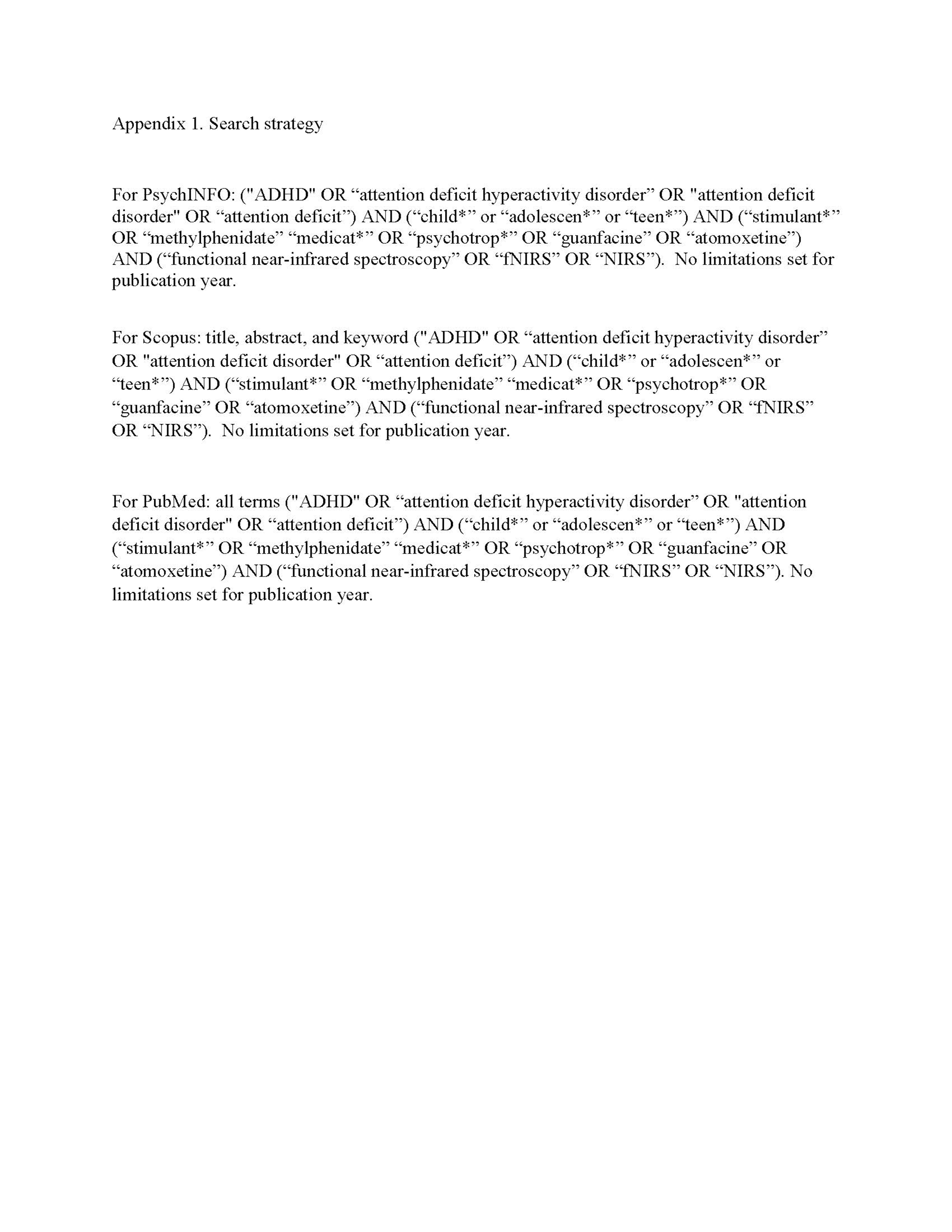

Supplement: Supplementary file 1 [file Image_1.jpg]
