# Supplementary material for: Using fNIRS to evaluate ADHD medication effects on neuronal activity: A systematic literature review
Source: Front Neuroimaging. Author manuscript; Available in PMC 2023 Apr 6. (PMC10078617; doi:10.3389/fnimg.2023.1083036)
Supplement: Supplementary file 1 [file NIHMS1881201-supplement-Appendix_1.pdf]

Appendix 1. Search strategy

For PsychINFO: ("ADHD" OR “attention deficit hyperactivity disorder” OR "attention deficit disorder" OR “attention deficit”) AND (“child*” or “adolescen*” or “teen*”) AND (“stimulant*” OR “methylphenidate” “medicat*” OR “psychotrop*” OR “guanfacine” OR “atomoxetine”) AND (“functional near-infrared spectroscopy” OR “fNIRS” OR “NIRS”). No limitations set for publication year.

For Scopus: title, abstract, and keyword ("ADHD" OR “attention deficit hyperactivity disorder” OR "attention deficit disorder" OR “attention deficit”) AND (“child*” or “adolescen*” or “teen*”) AND (“stimulant*” OR “methylphenidate” “medicat*” OR “psychotrop*” OR “guanfacine” OR “atomoxetine”) AND (“functional near-infrared spectroscopy” OR “fNIRS” OR “NIRS”). No limitations set for publication year.

For PubMed: all terms ("ADHD" OR “attention deficit hyperactivity disorder” OR "attention deficit disorder" OR “attention deficit”) AND (“child*” or “adolescen*” or “teen*”) AND (“stimulant*” OR “methylphenidate” “medicat*” OR “psychotrop*” OR “guanfacine” OR “atomoxetine”) AND (“functional near-infrared spectroscopy” OR “fNIRS” OR “NIRS”). No limitations set for publication year.
